# Effects of Intrinsic and Extrinsic Visual Cues on Consumer Emotion and Purchase Intent: A Case of Ready-to-Eat Salad

**DOI:** 10.3390/foods9040396

**Published:** 2020-03-31

**Authors:** Pitchayapat Chonpracha, Ryan Ardoin, Yupeng Gao, Pamarin Waimaleongora-ek, Georgianna Tuuri, Witoon Prinyawiwatkul

**Affiliations:** 1 School of Nutrition and Food Sciences, Louisiana State University, Agricultural Center, Baton Rouge, LA 70803, USA; pitchayapat_chonpracha@hotmail.com (P.C.); rardoi7@lsu.edu (R.A.); ygao19@lsu.edu (Y.G.); gtuuri@agcenter.lsu.edu (G.T.); 2 Institute of Nutrition, Mahidol University, Phutthamonthon Rd., Salaya, Phutthamonthon, Nakhon Pathom 73170, Thailand; pamarin.wai@mahidol.ac.th

**Keywords:** consumer perception, emotion, purchase intent, salads, visual cues

## Abstract

With increasing demand for ready-to-eat (RTE) fresh vegetables, it is important to understand how visual information cues, both intrinsic and extrinsic, affect consumer perception of these products. This study developed an emotional and wellness lexicon related to RTE salads. Subsequent questionnaires with images of salads were used to quantify consumer (N = 150) emotional and hedonic perceptions related to green color shade, shape/size of pieces, multicolor scheme, product name, and packaging. The different visual cues significantly impacted emotions and their intensities. Qualitatively, feelings of health and wellness predominated across salad samples. Negative emotions were more influenced by size of piece and green-color (intrinsic), while positive emotions were influenced by viewing salads of multiple colors (intrinsic) and packaging (extrinsic). Pale green salads were generally less liked than darker green ones. Values, in one case, ranged from 4.39 to 7.28 (on a 9-point hedonic scale), but naming the product (“iceberg lettuce”) did raise the lowest score to 5.75. The addition of vegetables with orange and purple colors to the salad mix had a positive impact on the perception of pale green salads. This study demonstrated that intrinsic and extrinsic visual cues significantly influenced consumer emotions, hedonic perception and purchase intent of RTE salads, but the effects of extrinsic cues were generally less prominent.

## 1. Introduction

Consumers use various intrinsic and extrinsic informational cues to form impressions about the quality of food products and to make subsequent purchase and consumption decisions. Intrinsic attributes are inherent to the product itself and cannot be manipulated without affecting its physical properties, such as appearance, taste and texture. In contrast, extrinsic sources of information are related to the product but are not physically a part of it, such as labeling, packaging, marketing information, or situational contexts [1,2]. In the product development process, both intrinsic and extrinsic cues can be directed toward generating positive sensory expectations, which can then dictate judgments of experienced quality upon consumption [3].

Sight is usually the first sense connected to our evaluation of foods [4]. While taste is reported to have the greatest influence on food choice [5], visual information is typically processed prior to food entering the mouth [6] and can even influence flavor perception [7]. These visual cues are not limited to intrinsic characteristics of the product itself (e.g., portion size, shape, color), but also pertain to extrinsic characteristics such as food packaging [4]. In addition to packaging design (e.g., color scheme, transparency/opacity, pictures), written information presented thereupon can also affect perceived value and expected outcomes, including how the product will be liked [8,9]. Ultimately, visual cues can be very impactful and influence consumer sensory expectations, hedonic evaluations, and emotions associated with food products and the overall eating experience [10,11,12,13,14].

Evaluating food-evoked emotions has recently gained interest in the fields of sensory and consumer research [15]. Emotional data have demonstrated added value, compared to traditional liking scores alone, in discriminating products, predicting food choice, and determining purchase intent. Furthermore, consumer emotional profiles are differentially affected by intrinsic and extrinsic product characteristics [10]. Visually, for example, food color intensity (intrinsic) and exposure to packaging (extrinsic) [16] have been shown to affect consumer product-related emotions and product liking [16,17]. Various methods for assessing food–elicited emotions have been employed [15], and the current study combined a few of these techniques to obtain emotional profiles for ready-to-eat salads based on visual evaluation.

The benefits of convenience and portability have driven the growing market for ready-to-eat (RTE) food products [18]. The demand for fresh–cut or minimally processed vegetable salads has also risen worldwide due to changes in demographics, lifestyles, and eating habits [19]. Thus, the hypothesis of this study was that visual intrinsic (green color shade, single vs. multiple vegetable colors, and size/shape of pieces) and extrinsic (product name and packaging) cues of RTE salads would affect consumer emotion expression (terms and their intensities), liking and purchase intent. The research experiment was divided into two main parts: (i) to develop an emotion lexicon related to RTE salads using the individual sample description technique and online questionnaire, and (ii) to investigate the effects of the above-mentioned intrinsic and extrinsic visual cues on consumer liking, emotion, and purchase intent of RTE salads. Data should shed light on salient emotions related to RTE salads, and guide methods to effectively employ visual cues in enhancing perceived product quality.

## 2. Materials and Methods

### 2.1. Development of Emotion Lexicon Related to Ready-To-Eat (RTE) Salads

Twelve RTE salads were purchased (from local supermarkets in Baton Rouge, LA, USA.) for evaluation. Selection was based on vegetable variety (e.g., iceberg lettuce, green cabbage, romaine lettuce, spring mix, and spinach), convenience orientation (complete salad kit with and without dressing), and packaging (elastic plastic bag and solid plastic container). The salads were stored under refrigeration (3–5 °C) one day before visual evaluations.

**Figure 1:**
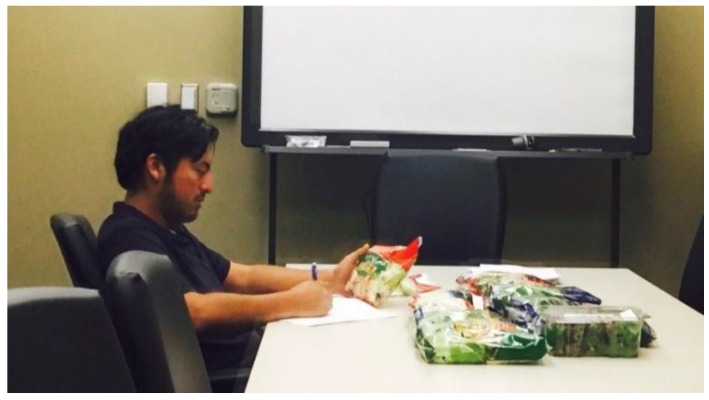
A consumer performing the modified individual sample description technique [20] with RTE salads.

Twenty-three consumers (Figure 1) who frequently purchased or consumed RTE salads (at least twice a week) were recruited to participate in the session. Emotion terminology was generated using a modified individual sample description technique [20]. Consumers were presented with the entire set of 12 salad samples at once but asked to evaluate them one at a time and write down perceived emotions associated with each salad. The typical total evaluation time was approximately 30–35 min (modified from [20]).

The selection of emotion descriptors began with sorting of terms within each salad. Terms with similar meaning were grouped together, and the most representative term from each group was chosen based on frequency of usage, and redundancies were eliminated. For example, if “safe” occurred more often than “secure” in relation to a salad sample, “safe” was retained. Then, emotion terms were pooled across all salad samples, and the same grouping and selection/elimination procedure was employed. This process yielded a list of 35 emotions pertaining to the RTE salads (Table 1).

These 35 terms were combined with those from the EsSense Profile^TM^ [21], an existing set of 39 common food-evoked emotions. A resulting list of 58 emotion terms was obtained after consolidating 16 duplicate terms (Table 2). In order to further identify those emotion terms most relevant to RTE salads, an online survey (Qualtrics, Provo, UT, USA.) was developed, and completed by an additional 118 consumers (consuming salads at least twice a week). This online survey presented consumers with photographs of RTE salads and asked them to select, in a check-all-that-apply (CATA) format, emotion terms (from a list of 58 terms) associated with the salads. The most relevant emotion terms, based on a selection rate of at least 30%, were chosen for rating measurement in the subsequent consumer study.

### 2.2. The Impact of Visual Cues on Consumer Liking, Emotions and Purchase Intent

#### 2.2.1. Visual Cues Tested in a Consumer Study

Photographs of RTE salads were captured, uploaded in an online questionnaire, and presented as the visual cues for the consumer study. These photograph images were employed as surrogates for fresh salads to maintain visual consistency [22] and to mitigate effects of sensory attributes other than appearance. The four visual cues of interest were: shade of green color, shape/size of vegetable pieces, single color or multicolor salad, and packaging (with or without) (see Table 3). The green color varied in visual green color shade to an extent deemed obvious to the normal human eye. From lightest to darkest green, the images depicted: square-cut iceberg lettuce (Sample A), shredded iceberg lettuce (Sample B), romaine lettuce (Sample C), or spinach (Sample D). The shape/size component was based on cut of the iceberg lettuce: square cut, considered large (square-L), or shredded, considered small (shredded-S). Additionally, the impact of product name (named or unnamed) was evaluated within these factors. Single-color salads consisted of lettuce only, and multicolor salad contained lettuce, shredded carrots (orange hue), and shredded red cabbage (purple hue). Multicolor salads were presented with and without their respective package. The visual effects of packaging presentation and multicolor were compared between pale green (PG; iceberg and romaine lettuce mix) and dark green (DG; romaine lettuce and spinach) salads.

#### 2.2.2. Consumer Study

Consumers were recruited from the Louisiana State University (LSU; Baton Rouge, LA, USA.) campus to participate in this study, which was approved by the LSU Agricultural Center Institutional Review Board (IRB HE#18-22). Selection criteria were that participants were regular salad consumers (at least twice per week) and over 18 years of age. Consumers (N = 150) completed the three questionnaires in 3 consecutive days. Using Compusense five^®^ Software (Compusense Inc., Ontario, Canada), online questionnaires were administered in partitioned booths under cool white lighting. Images of salads (Table 3) were presented in a randomized order. On day 1, the first questionnaire evaluated liking (a 9-point hedonic scale; 1 = dislike extremely, 5 = neither like nor dislike, 9 = like extremely) of green color and liking of size and cut for unnamed ready-to-eat salads. Each hedonic question was followed by a rating of emotions (a 5-point intensity scale; 1 = not at all, 2 = slightly, 3 = moderately, 4 = very much, 5 = extremely [21]; see justification for emotion term inclusion in Section 3.1). From each product image, purchase intent (PI) of the ‘actual product’ was evaluated on a ‘Yes’/’No’ scale. On day 2, the second questionnaire was administered with the only difference being that salads were named (i.e., “[square/shredded] iceberg lettuce,” “romaine lettuce,” or “spinach”). On day 3, the third questionnaire evaluated effects of single color, multicolor, and multicolor with packaging on liking of green color, liking of overall appearance, emotions, and PI for both PG and DG salads.

#### 2.2.3. Statistical Analysis

Analysis of variance (ANOVA with the Tukey’s HSD post-hoc test) and the Student’s *t*-test were used to determine whether mean liking and emotional ratings were significantly different at α = 0.05. In order to identify significant predictors for purchase intent, Logistic Regression Analysis (LRA) was used to model purchase intent as a function of liking and/or emotion intensity. These data were analyzed with SAS^®^ software (version 9.4, 2003). Correlation between emotion profiles and visual cues (green color, shape/size, multicolor and package) was demonstrated via a bi-plot by Principal Component Analysis (PCA). Emotion-driven green color liking scores were modeled using Partial Least Squares Regressions (PLSR), in which the standardized regression coefficient was used to further identify which emotions influenced liking scores. These analyses were carried out using XLSTAT^®^ software (Addinsoft Inc., 2015).

## 3. Results and Discussion

### 3.1. Development of Emotion Lexicon Related to Ready-To-Eat (RTE) Salads

Although the modified individual sample description technique [20] facilitated reporting of both positive and negative emotions, RTE salads seemed to elicit more positive than negative feelings (Table 1). The tendency for consumers to report more positive than negative associations with foods, or “hedonic asymmetry,” [23] has been previously reported and was not unexpected. In the present study, 26 of the 35 emotions identified from the individual sample description approach were considered positive, eight were negative, and the one remaining response “feel different” was neutral.

Specifically, the term “feel healthy” was repeatedly mentioned across samples. The idea of healthiness is often associated with the feelings of wellness, which is of interest to product developers. Wellness has been used to describe a subjective, multidimensional aspect of health, viewed with positive valence [15]. While the scientific literature has attempted to distinguish between the two concepts, it is not necessarily assumed that these differences are realized by all consumers, and for the purpose of this qualitative assessment the terms were considered separately (Table 1).

From the online CATA survey including 58 emotions (Table 2), nine were selected by at least 30% of the 118 participating consumers and used for further measurement in the subsequent consumer study. They were, in order of selection frequency: feel healthy, feel wellness, safe, satisfied, active, good, happy, interested, and refreshing. The terms “feel healthy” (79%) and “feel wellness” (58%) were the two most common responses and the only two reported by over half of the participants. Previous research of implicit color associations and emotional responses suggested that green color could elicit “energized” feeling [24], and using only food images (photographs), as in the present work, researchers demonstrated consumer capacity to experience “desire” for the actual product [22,25]. Therefore, these two emotions were included. Despite the hedonic asymmetry suggested from our emotion screening, the negative emotions “bored,” “disgusted,” “guilty,” and “worried” were also included based on their significant impact (typically negative) on purchase intent for various products with health benefits [26,27,28]. Although not reaching the 30% selection rate from the online questionnaire, the feeling “special” was also included due to its prominence in the open-ended individual sample description session. Accounting for these additional feelings would help depict a more comprehensive consumer perceptions related to RTE salads. As such, these seven additional emotion terms were incorporated for rating; hence, a total of 16 terms used for the subsequent consumer study.

### 3.2. The Effect of Visual Cues on Consumer Liking of Ready-To-Eat (RTE) Salads

Liking of “green color” and liking of “size of the cut pieces” were analyzed separately, based on combinations of green color shade and naming, or shape/size of pieces and naming, respectively (Table 4). Shade of green color had a significant (*p* < 0.05) impact on hedonic scores independent of whether names were presented with the images. For unnamed samples, the two darkest salads (Sample C and Sample D) were liked most in terms of green color (*p* < 0.05), with mean scores of 7.28 and 7.09, respectively. In practical terms, these mean ratings, above “like moderately,” for darker green salads indicated substantial hedonic superiority compared to Sample B (below the “like slightly” criterion) and the most pale Sample A, which was scored on the negative, or “dislike,” side of the scale (mean of 4.39). More intense green color may have implied greater freshness [29].

When salads were named, the darker “romaine” salad scored significantly higher (7.28) than both of the pale “iceberg lettuce” samples (5.75 and 6.0, Table 4). The only statistically significant (*p* < 0.05) effect of naming, however, was observed when comparing Sample A (iceberg lettuce but unnamed) with “iceberg lettuce” (named). Perception of this pale green color, which was “disliked” at face value, was boosted by identification of the vegetable (increase from 4.39 to 5.75). The effect of green color shade was slightly less pronounced when salads were named, but the darker green shades still clearly predominated in terms of hedonic judgments. 

When provided information about the salad constituent (iceberg lettuce, romaine lettuce, or spinach), consumers’ attentions may have turned to recollections of recent eating experiences and/or expectations of how the food would make them feel [30]. By calling upon these higher-level cognitive processes after product information was disclosed, green color cues may have subsequently become less salient in consumer evaluations. Additionally, consumers’ levels of familiarity with the salad samples (once the identity was made known) may have influenced the importance of extrinsic information on value judgments [31].

On the other hand, no statistically significant differences in size/cut liking between Square-L and Shredded-S salads were observed, whether or not they were identified as iceberg lettuce (Table 4). Liking of size of piece was, however, directionally higher for Shredded-S than Square-L salads (mean differences of 0.40 (unnamed) and 0.49 (named)) on the 9-point hedonic scale). With other food products, such as cut/sliced meats [32], small/large snack foods [33], and shaped pasta [34], consumer perceptions were largely influenced by size and shape. Based on the present results, it seems that the cut and size of iceberg lettuce was less influential to liking than the green color shade and product identification (naming) among adult regular salad consumers. However, to influence children’s consumption of vegetables, shape has shown to be important [35].

When evaluating pictures of single-color salads (green lettuce only), multicolor salads (lettuce, carrots, and red cabbage), and multicolor salads with package (see Table 3 for images), only the phrase “ready-to-eat salad” was used to describe the products. Liking of green color and overall appearance was measured, for both pale green (PG) and dark green (DG) lettuce variations (Table 5). With PG lettuce, liking of green color was significantly higher (*p* < 0.05 statistically; >1-point increase practically) for multicolor (with and without packaging) than single color salads; liking of overall appearance was higher for multicolor PG salad than both the single color and packaged items. This suggests that for pale green colored salads, such as those made with iceberg lettuce, color addition (here, orange and purple) had a positive visual impact on consumers. It has been suggested that, when balanced appropriately within the presentation, a variety of colors enhanced the attractiveness of a dish, in the same way it did with artwork [36].

Consistent with the previously discussed results comparing four shades of green salad (Table 4), single color DG salads were liked more (*p* < 0.05) in terms of green color and overall appearance than single color PG versions (Table 5). This was also the case for green color liking when packaging was included in the image. Differences in liking were minimized between multicolor PG and DG salads, suggesting that including vegetables with a variety of colors increased the salad’s appeal. In other studies, vegetables with higher chroma and vivid colors have been positively associated with freshness and quality, compared to dull colored vegetables [37,38,39].

For DG salads, directional but insignificant increases in both visual liking dimensions were observed (from single color, to multicolor, to multicolor with package). Overall, it may become more difficult to achieve statistical improvement in liking scores for DG than PG salads. Our evidence suggested visual appeal of DG salad varieties relies more on their intrinsic green color than extrinsic cues. Furthermore, sensitivity to detect differences is often reduced for highly acceptable products using the 9-point hedonic scale [40]. In this study, dark green salad varieties generally received relatively high liking scores, approaching or exceeding the “like moderately” mark.

### 3.3. The Effect of Visual Cues on Consumer Emotions of Ready-To-Eat (RTE) Salads

**Figure 2:**
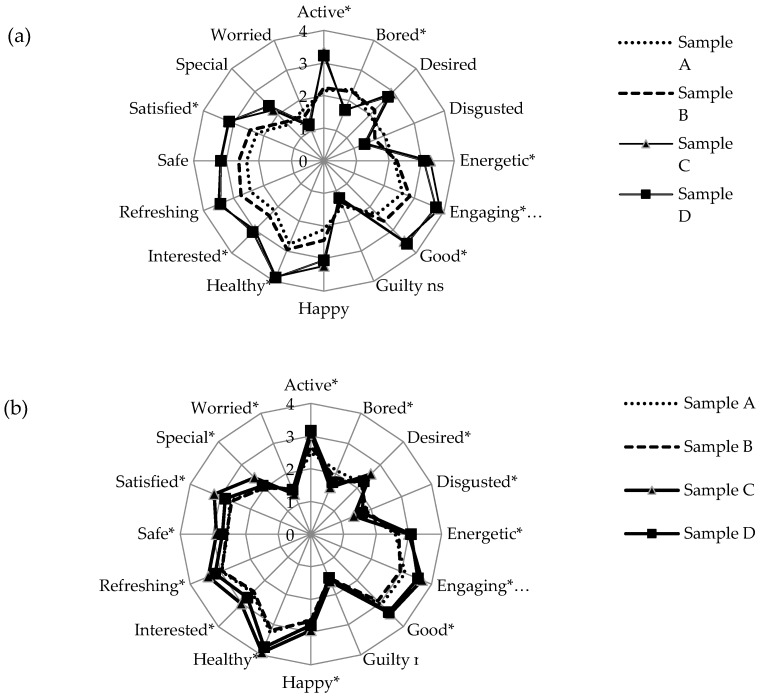
Mean emotion scores elicited by green color cues: (**a**) without product name and (**b**) with product name. * indicates significant difference among mean emotion scores (*p* < 0.05). Refer to Table 3 for sample (A–D) image and description.

Different shades of green color were not only important to visual liking, but also significantly (*p* < 0.05) affected consumer emotional reactions to images of salads presented without (Figure 2a) and with product name (the extrinsic information) (Figure 2b). Overall, darker green salads (samples C and D) elicited higher intensities of positive emotions (ranging from 2.20 to 3.88 on a 5-point intensity scale) than pale green salads (samples A and B, ranging from 1.56–2.95). Accordingly, the opposite was observed for negative emotions, with paler green salads generating scores ranging from 1.36 to 2.37 versus 1.13 to 1.69 for darker green. Eight of the measured emotions (active, bored, energetic, feel wellness, good, healthy, interested, and satisfied) showed statistically significant differences among salad samples. In fact, a difference of >0.3 units (meaning increased positive feelings and decreased negative ones) in the mean emotional intensity scores was observed between dark green and pale green samples across all eight emotion terms. Based on the current scale, these changes in effect can also be considered practically significant [41].

As previously mentioned, and substantiated in the current study, green color may be associated with memories and expectations about healthy foods, thereby influencing liking and emotions [16]. Interestingly, only consumers’ “guilty” feelings did not distinguish (*p* > 0.05) among salad samples (scores ranging between 1.20 and 1.50), although they did in similarly designed investigations of different product-types [17,27,28]. Perhaps the nature of the sample, green salads in this case, and their evocation of health and wellness overrode any expectations of guilt associated with consumption.

Presentation of salad name clearly affected emotional profiles (Figure 2a vs. Figure 2b), particularly for Sample A/iceberg lettuce (Figure 2b). Intensities of positive emotions (energetic, happy, healthy, interested, refreshing, satisfied and special) were all significantly higher for the named product-image. On the other hand, there was no effect on negative emotion intensity. As with liking scores, identifying the salad constituent by name may have elicited more specific memories and experienced emotions than the more general “ready-to-eat salad” label. For example, if experience dictated that iceberg lettuce is always pale, confirmed expectations may lead to more positive reactions. Previous research suggested that product naming had more impact on dieters than non-dieters [42], implying that health-conscious eaters were more sensitive to this external cue. In the present study, all participating consumers reported consuming salad at least twice per week (based on our initial screening), perhaps lending to the positive emotional effect of naming information.

**Figure 3:**
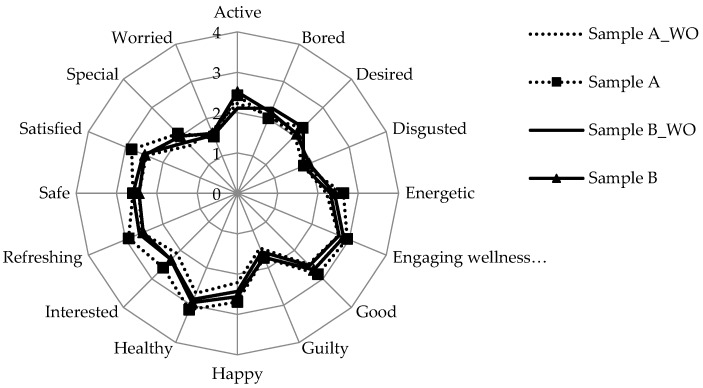
Mean emotion scores elicited by salad size, without (WO) and with product name. Refer to Table 3 for sample (A and B) image and description.

Comparing emotions related to size/shape of iceberg lettuce pieces (without inclusion of salad name), only healthy and wellness feelings proved to be significantly different (*p* < 0.05, Figure 3) between Square-L and Shredded-S salads, with the larger square-cut (Square-L) salads yielding higher intensity scores. When the vegetable name was given (iceberg lettuce), these two emotional intensities increased (*p* < 0.05). The same mechanism suggested above, for color-elicited emotions, is suspected. To formally differentiate between “health” and “wellness” is beyond the scope of this study and is an issue that is not fully resolved in the literature. However, health is sometimes viewed as a more objective measure of physical well-being, while wellness incorporates physical, emotional, and spiritual aspects [15]. In the present context of self-reported emotions, both are considered innately subjective, and it is not expected that consumers adhered to such formal definitions.

**Figure 4:**
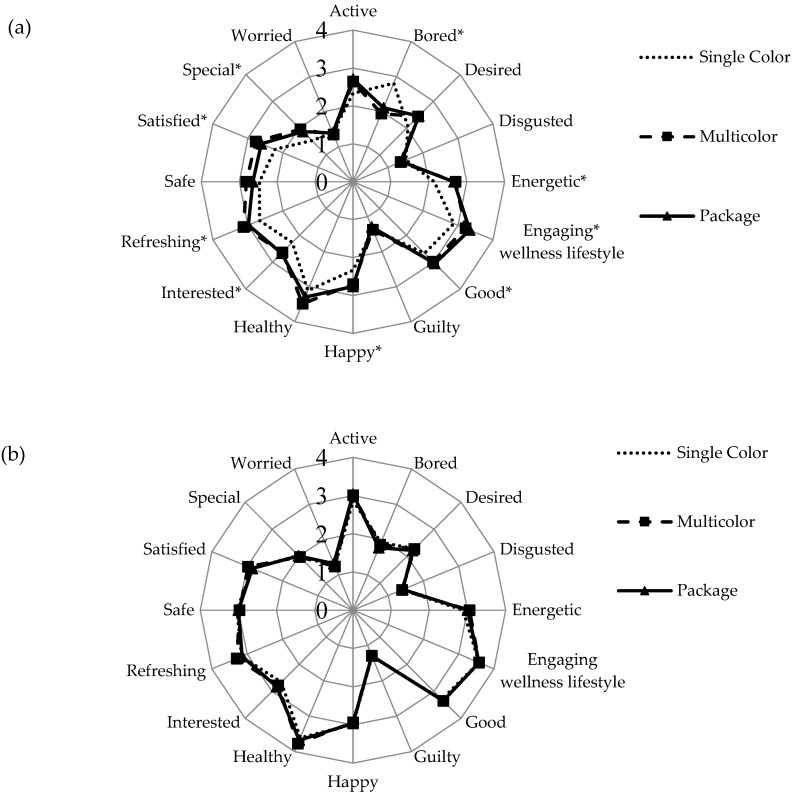
Mean emotion scores elicited by visual cues: single color, multicolor and package for (**a**) pale green color salad and (**b**) darker green color salad. * indicates significant difference among mean emotion scores (*p* < 0.05). Refer to Table 3 for sample image and description

Consumer emotions showed a similar trend to those of liking of overall appearance and green color as affected by monochromatic salads versus multicolor salads with and without package. Significant effects of multicolor and package on consumer emotions were observed for PG colored salads (*p* < 0.05) (Figure 4a) but not for DG salads (Figure 4b). Notably, the feeling “special” showed a mean increase of >0.4 units with addition of vegetables with orange and purple hues and package depiction, compared to the single-color PG sample. The single pale color also induced significantly more boredom (higher “bored” intensity) than its multicolor counterparts. Indeed, a meal consisting of a single color or only white foods was perceived as boring by some consumers [43]. As with liking scores, the high intrinsic acceptability of green color shade for DG salads may have left little room for further increasing emotional intensities from added color components or the package cue. It was suggested elsewhere [44] that intrinsic product characteristics are more associated with emotions than packaging, as was also observed here.

### 3.4. Correlation between Emotional Profiles and Visual Cues of Ready-To-Eat (RTE) Salads

**Figure 5:**
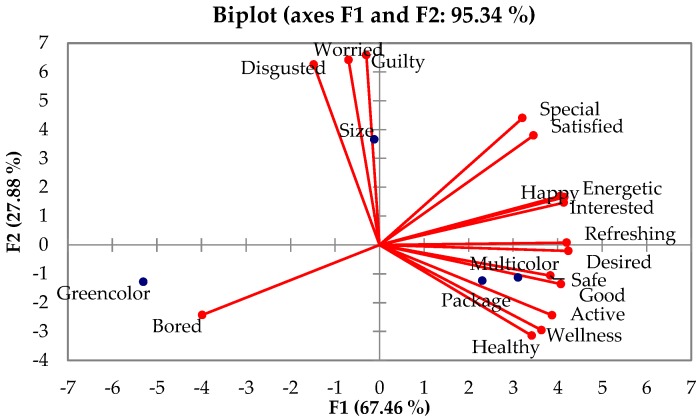
A PCA-biplot of emotion terms elicited by four visual cues (green color, size, multicolor and package) of ready-to-eat (RTE) salads.

Figure 5 shows the PCA bi-plot depicting correlations between the four visual cues (green color, size, multicolor and package) and emotion. Results indicated that the visual cues significantly influenced the emotion ratings, as both PC components can explain the variance up to 95.34%. The differences between positive and negative emotions were heavily seen in the first PC dimension, which accounted for 67.46% of the total variance. In the first dimension, negative emotions were on the left while positive ones on the right. The green color effect was correlated with the emotion “bored.” Conversely, most of the positive emotion terms were characterized by the multicolor and package cues. The second dimension accounted for 27.88% of the total variance. It can also be seen that the rest of the negative emotions (disgusted, guilty, and worried) were loaded on this dimension. These negative emotions were generally associated with the cut size of salads.

### 3.5. Correlation between Emotions and Consumer Liking of Green Color of Ready-To-Eat (RTE) Salads

**Figure 6:**
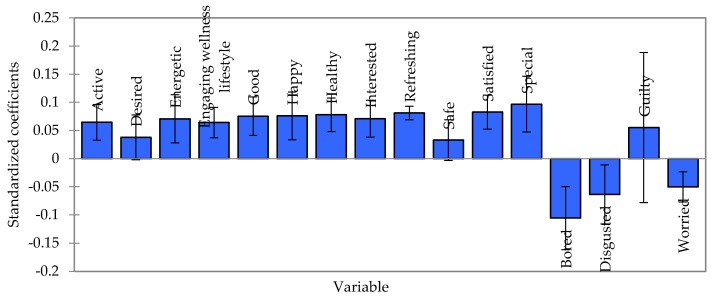
Correlations between emotional attributes evoked by green color cues and the liking of green color based on the partial least squares regression analysis (PLSR).

Figure 6 shows correlations between emotional attributes evoked by green color cues and liking of green color of salads at a confidence interval 95% using PLRS. It was observed that consumer liking scores for green color salads were driven by the positive emotions. The standardized regression coefficients loaded across all variables were 0.033 to 0.097. The emotion “special” showed the highest standardized regression coefficient value, whereas the term “safe” showed the lowest value. This implied that feeling “special” largely influenced liking scores of green color salads. In contrast, the negative emotions (bored, disgusted, and worried) contributed to decreased liking of green color of salads. As expected, the term “bored” with a standardized regression coefficient of −1.06 was an influential emotion associated with negative liking. Indeed, the liking score would decrease if consumers rated those negative terms with higher scores. Interestingly, the term “guilty” was positively correlated with green color liking scores. It is possible that consumers consciously considered health benefits of salad consumption and thus, would not feel guilty about consuming these products, even the pale green versions. Consequently, this term was positively correlated with green color of salads and did not decrease the liking scores.

### 3.6. The Effect of Visual Cues on Purchase Intent (PI) of Ready-To-Eat (RTE) Salads

The odds of positive PI (“Yes” response) were modeled separately based on liking and emotion scores for each of the four visual cues (green color, size, multicolor and package) (Table 6). A simple model using liking alone as a predictor showed a significant positive effect on PI (*p* < 0.05) from liking of size, multicolor and package. A one unit increased (on a 9–point hedonic scale) was associated with an increase in PI odds of 52%, 168%, and 171%, respectively. However, any effect of green color liking did not meet the criterion for significance (*p* = 0.5255).

When considering the nine emotional intensities as predictors of PI in a model, the impact of individual emotions depended on the eliciting condition under investigation. Results showed that increasing positive emotions would increase odds of positive PI, and more intense negative emotions would have the opposite effect (Table 6). The emotion “active” showed the most consistent significance for three of four visual cues (except shape/size), with each one-unit increase raising predicted PI odds by 2.5 to 5 times. The “disgusted” emotion scores for green color and multicolor would reduce PI odds by 85% and 78%, respectively, and a one-unit increase in “healthy” score was associated with a 3.8 to 5 times predicted rise in PI odds.

When including both liking and emotion in the model, green color and multicolor eliciting conditions became more significant predictors (*p* < 0.05). Further model selection techniques should be employed to obtain the most practical models. These results do, however, demonstrate the importance of the visual experience in food choice and purchase intent, influenced by both intrinsic and extrinsic information sources.

## 4. Conclusions

A multifaceted approach to qualitative lexicon development, as it pertains to the appearance of food products, may enhance the quality of consumer emotional profile data. The present combination of methods narrowed the most relevant emotions associated with RTE salads down from a list of 58 to 16 terms. Of these, several proved to distinguish among salad samples, depending on the prominent visual cues presented to consumers. Given the observed perceptions of RTE salads, future studies should elucidate between the product-evoked feelings of “healthy” vs. “wellness.” The effects of both intrinsic and extrinsic visual cues on emotion and liking were evident; however, color may be more important to positive product perceptions of salad than size of the salad pieces. The intrinsic value of a darker green color-shade invoked greater impressions of hedonic and emotional benefits for the salad consumers, repressing the potential for feelings of guilt and even overriding the marked improvement in consumer liking obtained by adding color variety to pale monochromatic salads. Our results suggested that liking of intrinsic visual characteristics of salads may moderate the effects of extrinsic cues. Providing additional product information such as naming or information on packaging may help reinforce positive tendencies toward making healthy food choices and purchase intent.

## Figures and Tables

**Table 1:** Emotion terms (35) elicited by ready-to-eat (RTE) salads, as generated by the modified individual sample description technique [20].

Emotion Terms		
Accomplished (+)	Excited (+)	Peaceful * (+)
Bored * (-)	Feel different (N)	Pleasant * (+)
Calm * (+)	Feel healthy (+)	Pleased * (+)
Comfortable (+)	Feel special (+)	Refreshing (+)
Confident (+)	Feel wellness (+)	Safe * (+)
Creative (+)	Fullness (+)	Satisfied * (+)
Curious (+)	Guilty * (-)	Steady * (+)
Dangerous (-)	Happy * (+)	Trust (+)
Desired (+)	Interested * (+)	Unique (+)
Disappointed (-)	Joyful *(+)	Warm * (+)
Discouraged (-)	Mad (-)	Worried * (-)
Disgusted * (-)	Nostalgic * (+)	

* indicates 16 terms shared with the *EsSense Profile*^TM^ [21]. ( ) indicates the emotion status; + = positive emotion, - = negative emotion, N = neutral.

**Table 2:** Percentage of emotion terms (58) elicited by ready-to-eat (RTE) salads and mentioned by consumers (N = 118).

Emotion Terms	Percentage	Emotion Terms	Percentage	Emotion Terms	Percentage
Feel healthy	79%	Loving	15%	Glad	4%
Feel wellness *	58%	Peaceful	15%	Affectionate	4%
Safe	45%	Joyful	14%	Darling	3%
Satisfied	44%	Bored	14%	Disgusted	3%
Active	39%	Calm	14%	Merry	3%
Good	37%	Understanding	13%	Tender	2%
Happy	31%	Accomplished	11%	Tame	2%
Interested	31%	Excited	10%	Nostalgic	1%
Refreshing	30%	Feel different	8%	Polite	1%
Pleased	28%	Free	8%	Dangerous	
Trust	27%	Mild	1%	Mad	1%
Confident	25%	Unique	7%	Quiet	1%
Desired	25%	Eager	6%	Wild	1%
Energetic	25%	Curious	6%	Aggressive	0%
Comfortable	24%	Disappointed	6%	Creative	0%
Feel special	23%	Guilty	5%	Discouraged	0%
Good–natured	22%	Adventurous	5%	Fullness	0%
Pleasant	22%	Enthusiastic	5%	Steady	0%
Friendly	21%	Warm	4%		
Worried	18%	Whole	4%		

* Also referred to as “engaging in a wellness lifestyle”.

**Table 3:** Salad images used to determine visual cue effects.

Visual Attribute		Ready-to-Eat (RTE) Salad Samples			
Unnamed	Lightness/Darkness	Sample A 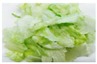	Sample B 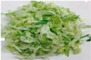	Sample C 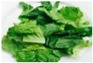	Sample D 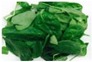
	Shape/Size	Square-L 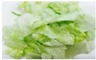		Shredded-S 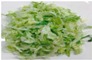	
Named	Lightness/Darkness	Iceberg Lettuce 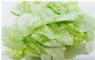	Iceberg Lettuce 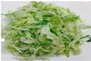	Romaine Lettuce 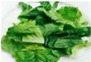	Spinach 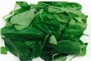
	Shape/Size	Square Iceberg lettuce 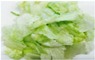		Shredded Iceberg lettuce 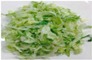	
Unnamed	Pale green (PG)	Single-color 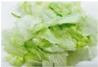	Multicolor 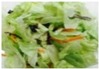	Multicolor with Package 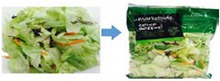	
	Dark green (DG)	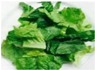	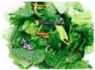	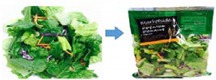	

**Table 4:** Mean liking scores of green color and size of ready-to-eat (RTE) salads.

Visual Cue Factors	Product Naming	RTE Salads *			
		Sample A	Sample B	Sample C	Sample D
Green color	No	4.39 c	5.40 b	7.28 a	7.09 a
	Yes	5.75 b	6.00 b	7.28 a	6.52 a,b
	*p*–value	0.000 **	0.07	0.961	0.074
		Square-L	Shredded-S		
Size	No	4.96 ns	5.36 ns	ND	ND
	Yes	5.27 ns	5.76 ns	ND	ND
	*p*–value	0.382	0.178		

* Sample A—Square Iceberg; Sample B—Shredded Iceberg; Sample C—Romaine; Sample D—Spinach; Square-L—square-cut iceberg lettuce (large piece); Shredded-S—shredded iceberg lettuce (small piece). a, b, c indicated significant differences of mean scores in each row (*p* < 0.05). ns indicated no significant differences of mean scores (*p* > 0.05). ** indicated significant differences of mean scores in each column using the Student’s *t*-test. ND not determined.

**Table 5:** Liking scores of overall appearance and liking scores of green color for ready-to-eat (RTE) salads with a single color, multicolor and package.

Visual Cue Factors	Liking of Green Color			Liking of Overall Appearance		
	PG *	DG *	*p* Value	PG	DG	*p* Value
Single color	5.49 b	6.92 ns	<0.0001 **	5.28 b	6.39 ns	0.001 **
Multicolor	6.65 a	6.93 ns	0.293	6.51 a	6.56 ns	0.829
Package	6.37 a	7.10 ns	0.029 **	6.24 b	6.68 ns	0.078

* PG: pale green lettuce, DG: dark green lettuce. (see Table 3 for images). a, b, c: indicated significant differences of mean scores in each column (*p* < 0.05). ns indicated no significant differences of mean scores (*p* > 0.05). ** indicated significant differences of mean scores in each row using the Student’s *t*-test.

**Table 6:** Predicting purchase intent of the ready-to-eat (RTE) salads by sensory visual cues.

Variables Used in Model	Sensory Visual Cues Effects							
	Green Color		Size		Multicolor		Package	
	*p*-Value	OR ^ˆ^	*p*-Value	OR	*p*-Value	OR	*p*-Value	OR
**Liking only**								
Liking	0.5255	0.933	0.0076	1.521	<0.0001	2.681	<0.0001	2.711
**Emotions only**								
Active	0.023	3.652	0.955	1.030	0.050	2.523	0.035	5.028
Desired	0.185	0.430	0.536	0.794	0.468	1.441	0.094	3.274
Energetic	0.730	1.243	0.599	1.319	0.090	0.360	0.753	0.805
Wellness	0.354	0.464	0.120	0.389	0.426	1.400	0.188	0.320
Good	0.348	2.324	0.096	2.883	0.171	0.440	0.214	3.411
Happy	0.908	1.079	0.874	0.913	0.347	1.767	0.029	0.130
Healthy	0.054	3.869	0.672	1.213	0.174	0.451	0.043	5.047
Interested	0.520	0.601	0.629	0.780	0.744	0.831	0.220	2.541
Refreshing	0.789	0.838	0.041	2.751	0.091	2.822	0.178	0.349
Safe	0.049	0.299	0.749	1.136	0.118	0.497	0.056	0.280
Satisfied	0.049	4.265	0.541	0.740	0.064	2.996	0.074	5.002
Special	0.637	0.721	0.500	1.401	0.691	1.191	0.620	1.462
Bored	0.565	0.832	0.214	0.566	0.488	0.766	0.095	0.481
Disgusted	0.034	0.148	0.555	1.337	0.034	0.219	0.951	1.062
Guilty	0.216	0.394	0.866	1.081	0.462	1.626	0.913	0.909
Worried	0.014	1.646	0.346	0.619	0.836	1.142	0.138	0.183
**Liking and emotions**								
Liking	0.280	1.256	0.222	1.354	0.009	2.994	0.026	7.686
Active	0.016	4.735	0.943	0.962	0.050	2.685	0.022	13.732
Desired	0.200	0.433	0.490	0.771	0.935	1.044	0.102	4.055
Energetic	0.914	1.071	0.700	1.230	0.480	0.611	0.903	0.897
Wellness	0.325	0.440	0.105	0.362	0.693	1.203	0.249	0.204
Good	0.358	2.291	0.113	2.817	0.090	0.329	0.431	2.495
Happy	0.927	1.063	0.814	0.869	0.943	0.951	0.045	0.097
Healthy	0.043	4.291	0.469	1.428	0.565	0.677	0.042	7.597
Interested	0.790	0.799	0.786	0.868	0.835	1.129	0.144	7.635
Refreshing	0.796	0.843	0.047	2.711	0.210	2.426	0.097	0.134
Safe	0.031	0.250	0.603	1.233	0.130	0.477	0.043	0.096
Satisfied	0.050	4.359	0.360	0.622	0.090	3.113	0.108	13.822
Special	0.480	0.603	0.553	1.357	0.641	0.788	0.466	0.472
Bored	0.695	0.879	0.272	0.592	0.684	1.213	0.847	1.154
Disgusted	0.023	0.123	0.385	1.589	0.070	0.215	0.145	8.596
Guilty	0.205	0.381	0.673	1.232	0.316	1.979	0.198	0.224
Worried	0.010	1.654	0.261	0.543	0.926	0.937	0.066	0.051

^^^ OR = odds ratio.
